# A case of dermatofibrosarcoma protuberans and reflectance confocal microscopy of a post‐surgical skin graft

**DOI:** 10.1111/srt.13117

**Published:** 2021-11-09

**Authors:** Shiri Nawrocki, Alexandra Rubin, Radhika Srivastava, Paola Chamorro, Babar K. Rao, Cindy M. Wassef

**Affiliations:** ^1^ Center for Dermatology Department of Pathology & Laboratory Medicine Rutgers Robert Wood Johnson Medical School Somerset New Jersey

**Keywords:** cutaneous sarcomas, dermatofibrosarcoma protuberans, reflectance confocal microscopy

## Abstract

Dermatofibrosarcoma protuberans (DFSP) is an overall rare malignancy yet is one of the most common cutaneous sarcomas. The diagnosis of DFSP is typically made following histopathologic examination of the lesion, classically revealing a storiform pattern of spindle cells with elongated nuclei infiltrating the dermis and subcutis. Surgical excision is the standard treatment. Local recurrence is estimated to occur in 20−50% of cases, thus frequent postsurgical monitoring is required. Noninvasive imaging modalities offer a potential alternative to multiple repeat biopsies. We report the first case where reflectance confocal microscopy accompanied clinical examination in monitoring for DFSP recurrence postsurgical excision.

DEAR EDITORS,

Dermatofibrosarcoma protuberans (DFSP) is an overall rare malignancy, but one of the most common cutaneous sarcomas.^1^ It commonly arises between the third and fifth decades of life,^3^ and presents on the trunk, or less frequently on the head, neck, or proximal extremities.^2^ It typically presents as an asymptomatic, slow‐growing, firm, skin‐colored, blue‐red or yellow‐brown, nodule or indurated plaque with irregular borders within the dermis or subcutis, and is typically freely mobile over deep structures.^1^ ^−^ ^3^ On histology, DFSP consists of a storiform pattern of spindle cells with elongated nuclei infiltrating the dermis and subcutis.^1^ The mitotic count is typically low.^1^ Immunohistochemically, the mass shows strong expression of CD34 and vimentin, but does not stain for factor XIIIa, cytokeratins, smooth muscle actin, S100 protein or desmin.^1^ Surgical excision is the standard treatment. Local recurrence is estimated to occur in 20−50% of cases, thus frequent postsurgical monitoring is required. Noninvasive imaging modalities offer a potential alternative to multiple repeat biopsies. We report the first case where reflectance confocal microscopy accompanied clinical examination in monitoring for DFSP recurrence postsurgical excision.

A 51‐year‐old woman presented for evaluation of an asymptomatic skin lesion on her left thigh. The patient first noted a dark brown patch at age 15 that occurred after a dog bite, which progressively changed in texture over time. Two years prior to presentation, the patient noted a new pink nodule within the original patch. Physical examination revealed a 3.0 cm × 3.0 cm × 1.25 cm erythematous, exophytic, firm nontender nodule surrounded by confluent hyperpigmented papules and nodules coalescing to form a 7.0 cm × 8.0 cm plaque on the left proximal anterior thigh (Figure 1). Histopathologic examination revealed a densely cellular spindle cell neoplasm with rare mitotic figures (Figure 2A). A storiform pattern of monomorphic fibroblast‐like cells was present within the dermis, sparing the papillary dermis, and extending into the subcutis (Figure 2B and C). Additionally, reactive hyperpigmentation of the basal layer was observed. The spindle cells stained strongly for CD34 but lacked expression of SOX10, S100, pancytokeratin or factor XIIIa (Figure 2D). A diagnosis of DFSP was made based on these findings.

**FIGURE 1:**
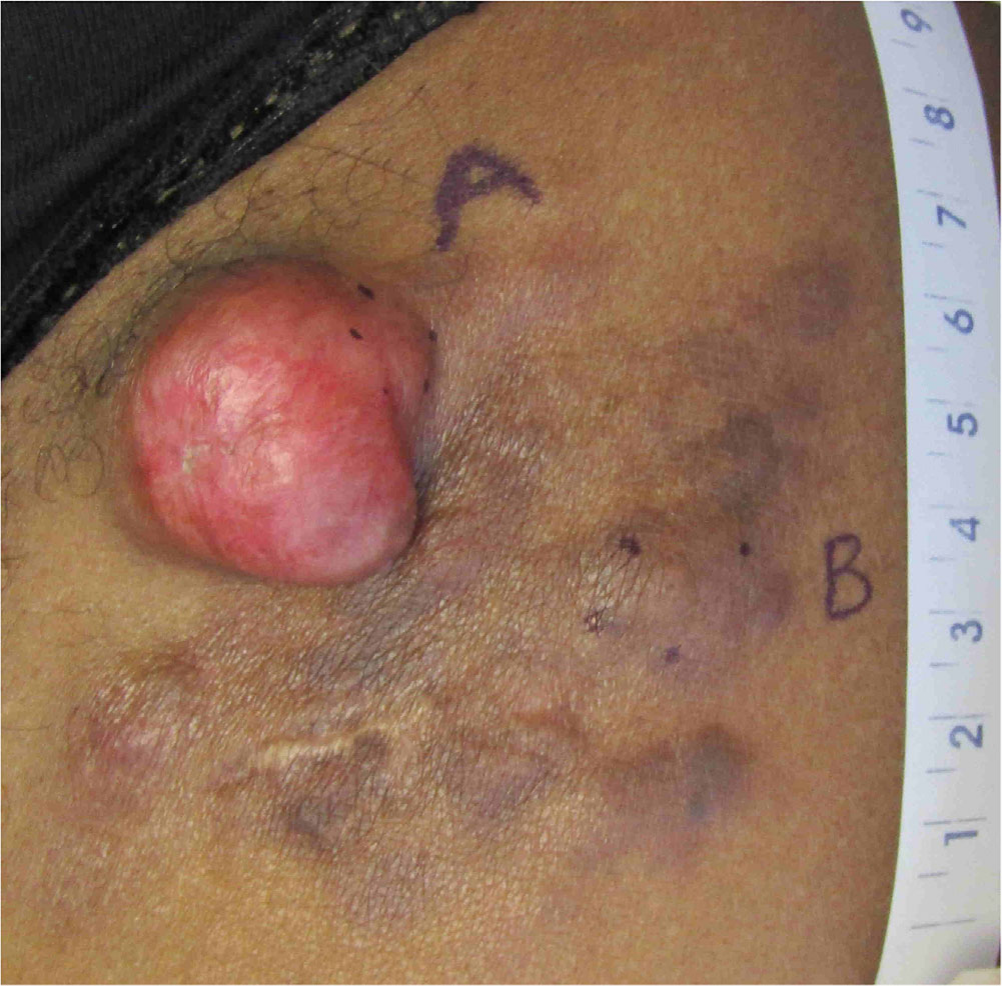
Examination revealed a 3.0 cm × 3.0 cm × 1.25 cm exophytic, erythematous nodule surrounded by confluent hyperpigmented papules and nodules coalescing into a 7.0 cm × 8.0 cm plaque on the left proximal anterior thigh

**FIGURE 2:**
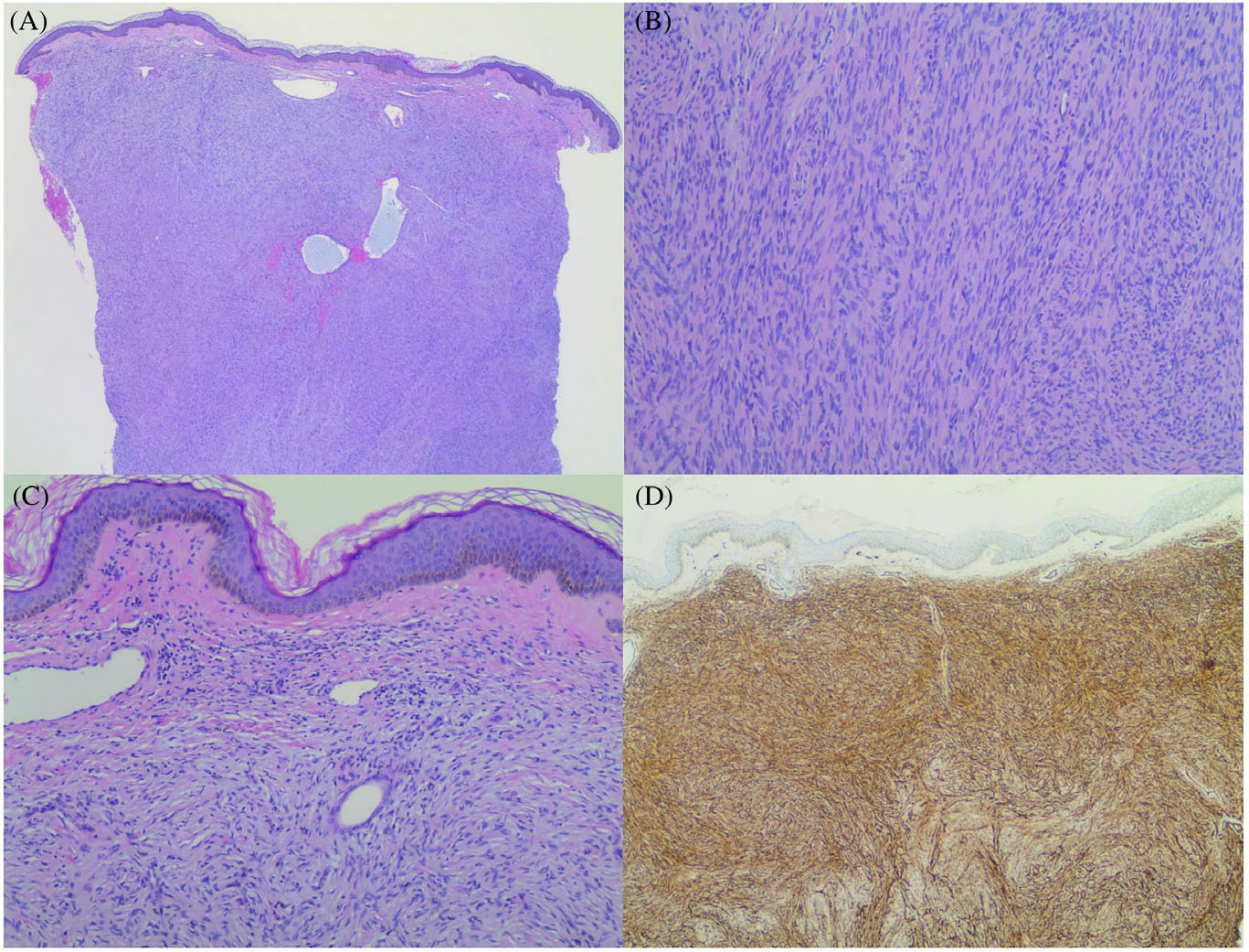
(A) Histopathology revealed a densely cellular spindle cell neoplasm (H&E, original magnification ×2). (B) There was a prominent storiform pattern of monomorphic fibroblast‐like cells with invasion of the subcutis (H&E, original magnification ×10). (C) Sparing of the papillary dermis with reactive hyperpigmentation of the overlying epidermis was seen (H&E, original magnification ×4). (D) The spindle cells stained strongly for CD34 (CD34 immunostain, original magnification ×4)

The patient underwent wide local excision of the tumor, requiring donor skin from the opposite thigh for full closure. Eight months following excision, a well‐healed, hyperpigmented 9 cm × 4 cm scar was observed with no recurrence on clinical exam. Reflectance confocal microscopy (RCM) was performed on the scar and its borders. Imaging of the skin graft revealed numerous hyperrefractile keratinocytes in all epidermal layers due to accumulation of melanin granules. Edged papillae were elongated at the dermal−epidermal junction and collagen fibers formed thick, parallel sheets in the papillary dermis (Figure 3). Spindle or stellate cells were not visible in the dermis of the skin graft, nor along the borders of donor and healthy skin.

**FIGURE 3:**
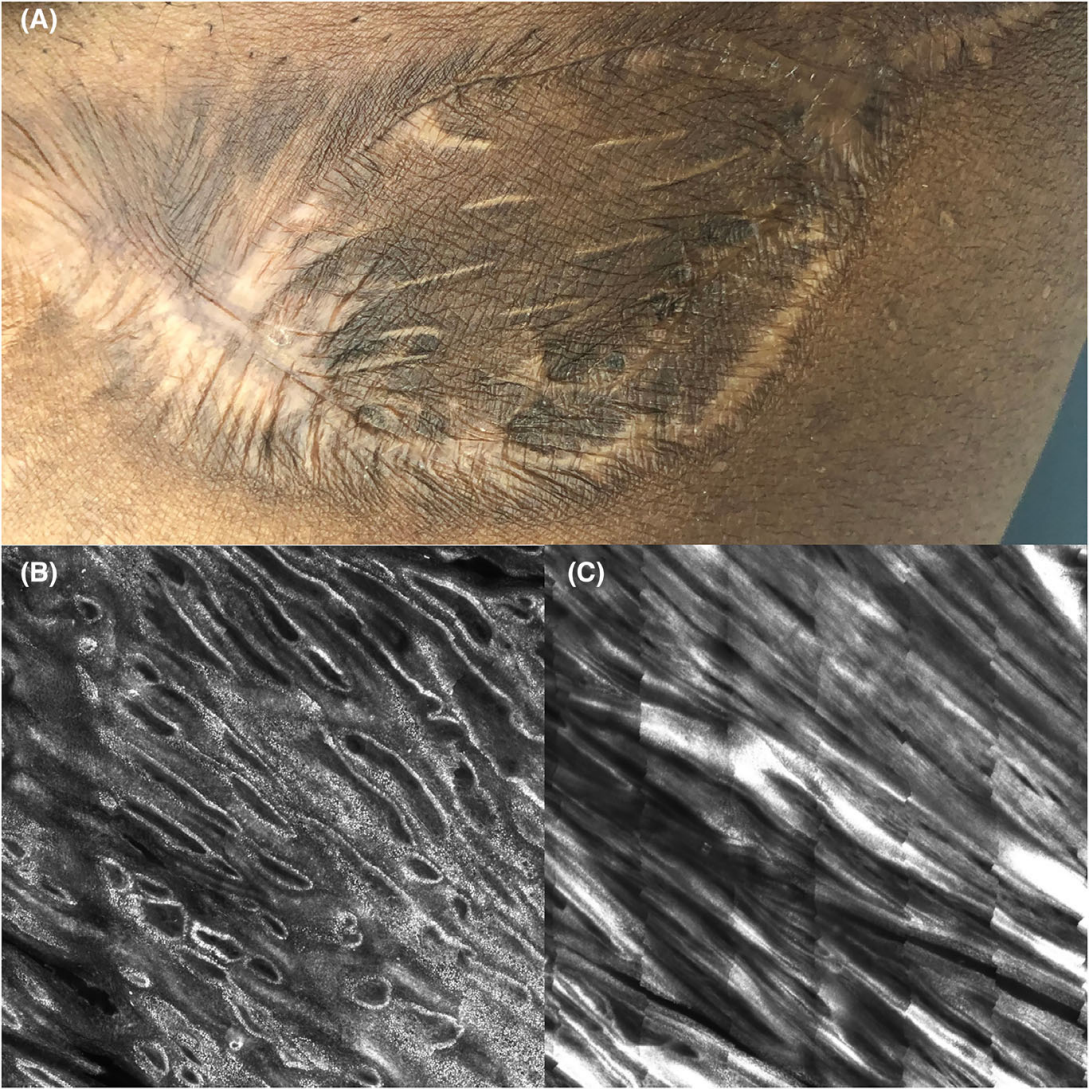
(A) Clinical image of the hyperpigmented scar following postsurgical excision of DFSP and skin graft placement. (B) RCM imaging shows elongated, edged rings surrounded by highly refractile, pigmented basal cells at the dermal−epidermal junction. The high refractive index likely resulted from postinflammatory accumulation of melanin granules. (C) Collagen has formed sheets of thick, parallel bundles in the papillary dermis. Stellate or spindle cells typically seen in DFSP are not visible

As an intermediate malignancy, DFSP has low metastatic potential, but is locally aggressive with a propensity to locally recur, often due to incomplete microscopic clearance of the tumor.^1^, ^2^ Surgical excision is the treatment of choice for DFSP. Given that DFSP has high rates of local recurrence (between 20% and 50%), DFSP may be treated with adjuvant radiotherapy if margins are equivocal or positive and frequent follow‐ups are required.^2^, ^4^

Noninvasive imaging modalities offer a potential alternative to multiple repeat biopsies. Two prior case reports have studied DFSP utilizing in vivo reflectance confocal microscopy (RCM). In these studies, DFSP was noted to have RCM features of focal regions of nonedged papillae at the level of the dermal−epidermal junction and bright‐elongated cells in the papillary dermis corresponding to spindle cells.^5^ A storiform pattern consisting of hyporefractile, or gray‐colored, neoplastic spindle cells intermingled with bundles of hyperrefractile collagen was also observed.^6^ The current case is the first utilization of RCM to visualize cellular‐level detail of a postsurgical scar and its borders following DFSP excision. RCM imaging resolution is limited to the superficial papillary dermis and would not capture deep invasion of neoplastic cells into the reticular dermis and subcutaneous fat. However, this imaging tool may be an option for initial noninvasive evaluation of new nodules or growths in initial conjunction with clinical exam. Of note, although features such as nonedged papillae and spindle cells were not observed in this case, disease recurrence below the superficial, papillary dermis cannot be definitively ruled out by RCM.

## CONFLICT OF INTEREST

B. K. Rao serves as a consultant for Caliber ID (Rochester, NY). The other authors declare no conflicts of interest.
